# Health literacy enhanced intervention for inner-city African Americans with uncontrolled diabetes: a pilot study

**DOI:** 10.1186/s40814-019-0484-8

**Published:** 2019-08-08

**Authors:** Hae-Ra Han, Manka Nkimbeng, Olayinka Ajomagberin, Kelli Grunstra, Phyllis Sharps, Susan Renda, Nisa Maruthur

**Affiliations:** 1 School of Nursing, The Johns Hopkins University, 525 N. Wolfe St., Room 526, Baltimore, MD 21205 USA; 2 Center for Community Innovation and Scholarship, The Johns Hopkins University, Baltimore, MD 21205 USA; 3 School of Medicine, The Johns Hopkins University, Baltimore, MD USA

**Keywords:** Type 2 diabetes, African American, Numeracy, Self-management, Glucose control

## Abstract

**Background:**

Disparities in diagnosis and control of type 2 diabetes mellitus are most evident in African Americans (AAs) with lower socioeconomic status. Health literacy is an important predictor of adequate self-management and control of diabetes. The purpose of this pilot study was to test the feasibility and preliminary efficacy of a health literacy-enhanced diabetes intervention, PLAN 4 Success (Prevention through Lifestyle intervention And Numeracy)-Diabetes, in inner-city, low-income AAs with uncontrolled type 2 diabetes.

**Methods:**

Nineteen of 30 participants who completed the baseline survey received the study intervention which consisted of 4-week health literacy training and disease knowledge education followed by two home visits and monthly phone counseling for over 24 weeks.

**Results:**

A retention rate of 58% was achieved at 24 weeks. All participants who completed the follow-up assessment at 24 weeks reported high satisfaction with the intervention. Participation in the PLAN 4 Success-Diabetes was associated with improved glucose control and psychological outcomes at 12 weeks but the positive trend was attenuated at 24 weeks.

**Conclusions:**

The current intervention protocols were in general feasible and highly acceptable. The results support health literacy training as a promising component of interventions to promote glucose control among inner-city AAs. Some changes are suggested to optimize the protocols, before conducting a randomized controlled trial. Future interventions should consider addressing social determinants of health such as transportation as part of designing an intervention targeting low-income AAs with uncontrolled type 2 diabetes.

**Trial registration:**

ClinicalTrials.gov, NCT03925948. Registered on 24 April 2019—retrospectively registered.

## Background

Type 2 diabetes mellitus (T2DM or diabetes hereafter) is an increasingly prevalent and serious health problem in the USA, costing the nation $327 billion annually [1]. African Americans (AAs) are particularly affected by diabetes. The age-adjusted prevalence of diabetes for AAs is 13.4%, nearly double the prevalence of non-Hispanic whites (7.3%) [1]. Further, AAs suffer higher rates of adverse consequences compared to whites, including heart disease and stroke (2–4 times more likely), blindness (50% more likely), kidney disease (2.6–5.6 times more likely), amputations (2.7 times more likely), and premature mortality (2.1 times higher) [2].

Limited health literacy—“the degree to which individuals have the capacity to obtain, process, and understand basic health information and services to make appropriate health decisions” [3]—is important to consider in the context of these racial disparities in diabetes. In the 2003 National Assessment of Adult Literacy, nearly one quarter (24%) of AAs had below basic health literacy compared to 9% of white Americans [4]. Limited health literacy was also more likely among those with less educational attainment and those receiving public insurance [4].

Limited health literacy is thought to impact health outcomes through multiple mechanisms, including decreased access and utilization of health care, sub-optimal provider-patient interactions, and lack of condition-specific appropriate self-care [5], all of which are crucial to diabetes management. In particular, limited numeracy skills—one’s ability to use quantitative information—have been associated with inadequate self-management among persons with diabetes [6–10]. While provider-patient communication is increasingly recognized as a critical component of the care process, AAs with limited health literacy may be unable to effectively communicate with their healthcare providers and frequently do not understand treatment recommendations [3, 10]. Further, they rarely question or discuss their treatment regimen with their care provider in detail [10]. AAs with limited health literacy may experience complications from their medical regimen, adjust their medication dosages on their own, or skip doses or take less than the prescribed dose, behaviors which put them in danger of not controlling their glucose [7]. Limited health literacy has also been associated with lower self-efficacy for chronic disease management among racial/ethnic minorities [8, 11]. Promising innovations for addressing culturally specific health literacy needs should be considered for the benefit of low-income AAs who suffer disproportionately from inadequate diabetes care [1, 2].

While evidence supports the link between culturally sensitive health education and the reduction of hemoglobin A1c (HbA1c) [12]—the gold standard measure of glucose control—many studies are limited by design such as failing to measure HbA1c [13–15]. Further, even though studies have targeted low-income AAs, they often do not address cultural and contextual considerations (e.g., health literacy) resulting in sub-optimal outcomes [16]. Few studies have examined the impact of allaying the effects of low health literacy on health outcomes or tailored interventions to increase health literacy levels of the participants. Recent randomized controlled trials (RCTs) showed that providing patients with health literacy skills training was highly effective for promoting healthy behaviors such as cancer screening [17] and physical activity [18] among individuals with limited health literacy. The findings suggest the types of strategies we should work toward for prevention of complications and on-going support for diabetes among low-income AAs. A RCT is required to evaluate the efficacy of health literacy focused intervention approaches in the management of diabetes among AAs. This was the basis for developing PLAN 4 Success-Diabetes (Prevention through Lifestyle intervention And Numeracy-Diabetes).

Using a community-engaged approach, we developed a health literacy-enhanced diabetes self-management intervention program for inner-city, low-income AAs, PLAN 4 Success-Diabetes. This approach to participatory research has been shown to be effective in informing culturally tailored interventions in minority populations [19]. To evaluate the feasibility, acceptability, and preliminary efficacy of the intervention, we conducted a pilot study with 24-week follow-up, as part of the preparation for a RCT. Specifically, the primary aim was to evaluate whether PLAN 4 Success-Diabetes was feasible and acceptable, and indicate attrition rates. Secondly, the aim was to evaluate changes in the glucose and psychosocial outcomes following the intervention. We hypothesized that participation in the PLAN 4 Success-Diabetes intervention would be associated with a reduction in glucose outcomes and improvements in psychosocial variables.

## Methods

### Design and sample

We used a single-arm pre- and post-test design for this pilot study in which we assessed the feasibility, acceptability, and preliminary efficacy of the PLAN 4 Success-Diabetes in inner-city AAs in Baltimore, Maryland. Community-dwelling AAs were recruited via referrals from inner-city federally qualified health clinics. Eligible participants were (1) aged 18 years or older, (2) residing in Baltimore, and (3) had uncontrolled diabetes (defined as HbA1C > 7%).

### PLAN 4 Success-Diabetes

The study intervention—PLAN 4 Success-Diabetes—consisted of four 1-to-1 ½-h weekly health literacy training and disease knowledge education sessions for 4 weeks (four in-person sessions), followed by two home visits and monthly phone counseling for over 6 months (five phone sessions). The intervention is theory-driven and builds on von Wagner’s model [20] to incorporate key elements such as health literacy, disease knowledge, and self-efficacy for better glucose outcomes.

The PLAN 4 Success-Diabetes was developed in partnership with AA members with diabetes from the targeted low-income, urban community to explore preferences for desired intervention components and outcomes. Initial intervention materials and approaches were reviewed with the Community Research Advisory Council at the Johns Hopkins Institute for Clinical and Translational Research which included representatives from AA serving community organizations, faith community leaders, AA patients with diabetes and caregivers, clinicians, and researchers. Modifications suggested (e.g., adding patient-provider communication activities in the weekly education program, incorporation of home visits in the follow-up protocol) were incorporated throughout the intervention development process. Participants were asked to join weekly face-to-face group education sessions over 4 weeks focused on health literacy skills training and disease knowledge education (causes and diagnosis of diabetes and steps to control diabetes, co-morbid conditions such as hypertension, Table 1). Weekly education sessions used role-plays to practice health literacy skills (e.g., reading food labels, medication slips) and visuals and colors to highlight key medical terminology in diabetes care, in addition to role-playing scenarios focused on patient-provider communication. Per recommendation by the community advisory committee, each weekly session also included activities to promote oral health literacy for effective patient-health provider communication. Education sessions were delivered by a trained nurse at local community health centers from which the study participants were identified and referred.

**Table 1:** Main topics for each weekly education session

Session	Content
1	• Types of diabetes • Risk factors for diabetes • Symptoms of diabetes • Consequences of inadequate management/treatment of diabetes • Strategies to maintain health
2	• Health screening and appointments • Communicating with healthcare providers • Management of hypo/hyperglycemia • Diabetes treatments and management
3	• Carbohydrates and blood glucose • Carb counting • Meal planning guidelines • Eating out
4	• Getting active • Exercising safely • Emotional health • Dental care

Two home visits were made by a trained nurse/research staff team, one within 1–2 weeks after the last education session, and another between the third and fourth phone counseling visit. The trained nurse assessed the patient’s home safety (clutter, fall prevention); food pantry; diabetic-friendly (reading food and medication labels); and neighborhood environment to identify barriers to and facilitators of physical activity, and to develop a plan to promote healthy eating and physical activity within that environment (e.g., increasing steps by using stairs, walking in a neighborhood school), in addition to assessing medication-taking and self-monitoring behaviors. The home visits were a strategy suggested by the community advisory committee to help participants manage their diabetes by making the best use of resources they could access. In close collaboration with the nurse, our participants worked to develop an individualized plan to prioritize which foods could be included in their dietary plan. Home visits also offered a comfortable setting and opportunity to revisit health literacy and other critical disease management skills (e.g., use of glucometer and blood pressure monitor) taught in the group format as necessary. At the second home visit, the team tracked the participant’s progress toward their goals for physical activity, healthy diet, medication adherence, and self-monitoring of glucose and blood pressure.

Finally, trained research staff provided individually tailored support via monthly counseling. The goals of monthly counseling were (1) to help each patient reach the individualized goal set during the nurse home visit; (2) to check on home glucose and blood pressure values; (3) to assess self-efficacy in managing diabetes and other life adversities; and (4) to work with the patient on strategies to manage the identified problems and provide referrals for medical equipment, fresh food products, and further care as needed.

### Procedures

Upon completion of baseline assessment, education sessions were delivered to groups of two to four persons. The education sessions lasted 60–90 min. Follow-up with the participants was performed with a phone call within 1–2 weeks from the completion of education and monthly thereafter for 6 months. At each phone counseling session, a counselor checked and discussed the participant’s progress toward his/her goals for physical activity, healthy diet, medication adherence, and self-monitoring of glucose and blood pressure, and the counselor answered questions or concerns about diabetes management. Nurse home visits were organized by the nurse and participant. Trained study staff collected data at baseline, 12 weeks, and 24 weeks from the start of the intervention by face-to-face interviews at a community health center. Participants received $20 at each data collection visit. Participants also received an additional $5 per education session to cover transportation expense.

### Ethical approval and consent

The study protocol was approved by the Johns Hopkins Medicine Institutional Review Board (IRB #00061339). All participants provided written informed consent prior to inclusion in the study.

### Outcomes

We collected data on study recruitment and retention, attendance at education sessions, home visit rates, and phone counseling completion rates. Feasibility was measured by the overall recruitment level and retention rate for the study. Acceptability was measured using a satisfaction survey developed for the purpose of this study. The secondary outcome measures included HbA1c, fasting glucose, health literacy, and psychosocial variables (diabetes knowledge, self-efficacy, social support, and depression), and quality of life, specifically the mean and standard deviation for these measure in the target population.

#### Feasibility and acceptability measures

The attrition rate was the total proportion of participants who received any portion of the study intervention but left the study before completing the final assessment at 24 weeks. We aimed to achieve an attrition rate of less than 40% for progression to a full RCT. Components of the satisfaction survey as the acceptability measure included self-reported satisfaction with the intervention program and delivery of its content (e.g., pleased with what I learned, educational materials presented in an engaging manner, program ran smoothly), responsiveness to questions and concerns, benefit from the intervention and study materials (program met expectation), and utility of knowledge and skills in managing diabetes given the program participation (will use knowledge and skills gained).

#### Study measures

Sociodemographics and medical characteristics were assessed at baseline using a study questionnaire. Study variables were measured at baseline, 12 weeks, and 24 weeks from the start of the intervention. Data were collected on the following outcomes: HbA1c, fasting glucose, health literacy, psychosocial variables (diabetes knowledge, self-efficacy, social support, and depression), and quality of life. The study nurse performed venipuncture to assess HbA1c, fasting glucose and lipids and used the A&D UA-767 device (A&D Company, Ltd, Tokyo, Japan) to measure blood pressure after the participant had been seated for 5 min [21]. The second and third blood pressure readings were averaged to obtain the mean BP.

Health literacy was measured by two instruments: Literacy Assessment in Diabetes (LAD) [22] and the Newest Vital Sign [23]. The Literacy Assessment in Diabetes (LAD) has high reliability and validity indices [22]. The items on the LAD are scored as correct/incorrect, with total possible scores ranging from 0 to 60. Higher scores indicated higher health literacy levels. The Newest Vital Sign [23] consists of four items and measures numeracy. After reviewing a nutrition label, participants are asked to answer questions based on some calculation of the nutritional information (e.g., fat, sodium) presented on the label. Total possible scores range from 0 to 4, with one point assigned for each correct response.

Diabetes knowledge and diabetes self-efficacy were measured with the validated Diabetes Knowledge Test [24], and Stanford Diabetes Self-Efficacy scale [25]. The Diabetes Knowledge Test assesses diabetes knowledge, medications, diet, and management with questions such as “What effect will an infection most likely have on blood glucose.” [24]. Correct responses are given a score of one. The Stanford Diabetes Self-Efficacy scale assesses participant’s efficacy in managing diabetes and maintaining healthy lifestyles. The scale asks how confident participants are in managing different tasks such as eating meals every 4–5 h every day or breakfast every day measured on a Likert scale of 0 “not at all confident” to 10 “totally confident.” Participant scores were the means across all items in the instrument [25].

A diabetes self-care index was created for this study. This index included seven questions on smoking, alcohol consumption, meal planning, consumption of high-fat foods, consumption of high sugar foods, consumption of sodium, and medium or high intensity exercise. These questions were coded into dichotomous responses with participation in healthy behaviors coded as “1” and active participation in negative health behaviors coded as “0.” A summary score for the index could range 0–7 and higher scores indicated better self-care.

We used modified Medical Outcomes Study-Social Support Survey (mMOS-SS) [26] to assess social support. mMOS-SS is a shorter version (10 items) of the MOS-SS which includes 19 items. The original version was used to measure social support in community-dwelling chronically ill persons. mMOS-SS covers emotional and instrumental domains of social support with strong evidence of reliability and validity [26]. Example items include “How often is someone available to take you to the doctor if you need it” or “How often is someone available who understands your problems?” Response options are from all of the time (5 point) to none of the time (1 point) with higher scores indicating higher levels of social support (total score range = 10–50).

The Patient Health Questionnaire (PHQ)-9 [27] addresses the severity of depressive symptoms based on the Diagnostic and Statistical Manual of Mental Disorders, fourth edition (DSM-IV). The PHQ-9 has nine items scored from 0 (not at all) to 3 (nearly every day) with total scores ranging from 0 to 27. Individuals are asked whether they have experienced any symptom(s) over the past 2 weeks. Evidence of reliability, validity, sensitivity, and specificity has been reported in community samples [27].

Quality of life was measured with the EuroQol (EQ) quality of life scale [28]. It contains the EQ-5D-3L that provides a single index of health status. The EQ-5D-3L assess five domains: mobility, self-care, usual activities, pain/discomfort, and anxiety/depressions. Responses show if participants do not have difficulty, have some problems, or have severe difficulty within the domains. Summary scores for the sample were created across each of the five items (range 0–2) with higher scores indicating better quality of life. A component of this scale is the EuroQol visual analog scale (EQ-VAS) which participants can use a number from 0 to 100 to identify their health state.

### Analysis

We used descriptive statistics to summarize sample characteristics and study variables. Specifically, we used the mean and standard deviation to summarize continuous variables. Categorical variables were summarized by frequency. Final analysis was performed using data from 11 participants who completed all data points. The main preliminary efficacy outcomes of interest were changes in HbA1c and fasting glucose. Effect sizes were calculated using the mean change from baseline to 12-week follow-up and from baseline to 24-week follow-up, each divided by the baseline standard deviation [29].

## Results

### Recruitment and retention

**Fig. 1:**
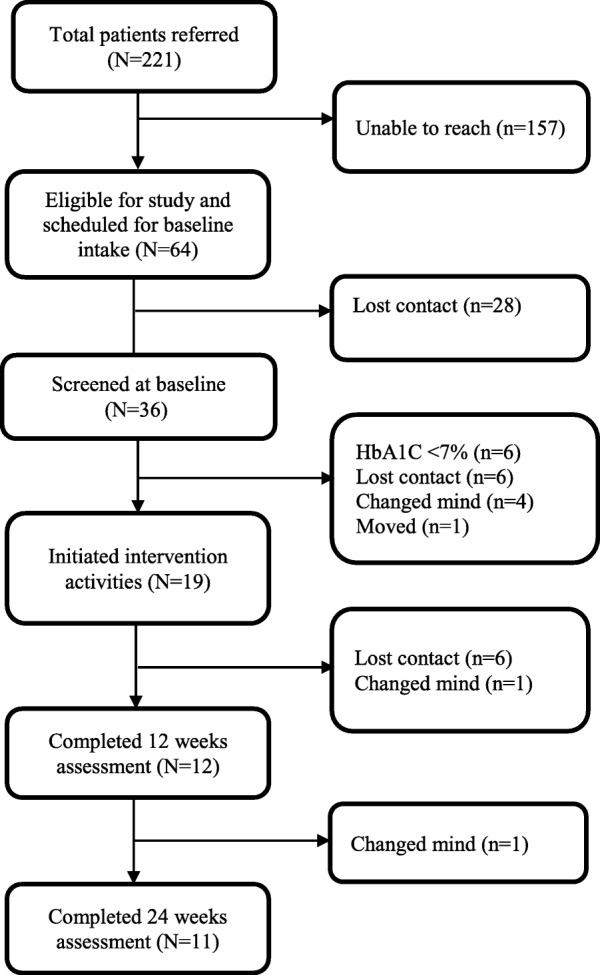
Recruitment and participant tracking

The number of patients referred and assessed for eligibility and the number of complete datasets assessed are presented in the CONSORT chart in Fig. 1. A total of 221 individuals were referred and 64 were scheduled for eligibility verification appointments. Of these individuals, 36 attended the appointments, and 30 were confirmed eligible. A total of 30 eligible participants completed the study assessment at baseline. Of those who initially agreed to participate in our study and completed the baseline assessment, 11 dropped out before the intervention began for reasons including lost contact (*n* = 6), changed mind (*n* = 4), and moving out of state (*n* = 1). As a result, our intervention was delivered to a total of 19 participants.

Table 2 shows the characteristics of the 19 participants who received the study intervention. Mean age of the 19 participants was 54 years. More than half of the sample (63.2%) were female and had high school diplomas and above (68.4%). The majority (57.9%) had insurance through Medicare and/or Medicaid. A little over a quarter of the sample was employed (26.4%) and 36.9% reported that it was difficult or very difficult to manage on their current income. The majority (94.7%) of participants reported having comorbidities (hypercholesterolemia, hypertension, heart disease, or asthma). All of the 19 participants were on medications for diabetes and most (89.5%) had primary care providers. Of those, eight discontinued their participation before the final data collection assessments at 24 weeks and were done, yielding 11 in the final sample (attrition rate = 42% at 24 weeks). There were significant differences in health literacy scores between the participants who completed the study (*n* = 11) and those who did not (*n* = 8). Specifically, participants who completed the study had higher scores on the Literacy Assessment in Diabetes (mean = 59.5 vs. 55, *p* < 0.001) at baseline. In contrast, participants who did not complete the intervention tended to be older (57.3 vs. 51.5) and more depressed (PHQ-9 mean score 10.3 vs. 6.4) and had less diabetes knowledge (Diabetes Knowledge Test mean score 5.2 vs. 6) and higher HbA1c (10.7 vs. 9.3) at baseline, but none of these differences were statistically significant.

**Table 2:** Sample characteristics at baseline

Variable	Mean (SD)^a^ or % for the total sample (*N* = 19)	Mean (SD) or % for the analytical sample (*n* = 11)	Mean (SD) or % for the dropout sample (*n* = 8)
Age, years	54.1 (8.8)	51.5 (10.4)	57.3 (5.6)
Female	63.2	72.7	50.0
< high school	31.6	27.3	37.5
Employed (full or part-time)	26.4	54.6	25.0
Difficult to manage with current household income	36.9	27.3	50.0
Insured (Medicare and/or Medicaid)	57.9	63.6	50.0
Comorbidity	94.7	90.9^b^	100.0^c^
On diabetes medication	100.0	100.0	100.0
Have primary physician	89.5	100.0	75.0

^a^Standard deviation

^b^Hypercholesterolemia (*n* = 7), hypertension (*n* = 6), heart disease (*n* = 2), asthma (*n* = 2)

^c^Hypercholesterolemia (*n* = 6), hypertension (*n* = 6), heart disease (*n* = 2)

### Acceptability

In terms of acceptability of the study intervention, we established this as acceptable to participants. The size of face-to-face education classes ranged from two to four participants per session. Seven of 11 participants who completed the final assessment missed at least one of the prescheduled education sessions but made this up at the following session or during one of the home visits. Participants completed on average one home visit (range = 0–2) and on average about three phone counseling sessions (range = 1–5). One-hundred percent of the final sample would recommend the program, with an overall satisfaction rating of 9.5 on a 10-point scale. Specific participants’ satisfaction ratings with the intervention delivery and utility were also high: pleased with what I learned (agree/strongly agree = 100%); educational materials presented in an engaging manner (agree/strongly agree = 100%); program met expectation (agree/strongly agree = 100%); and will use knowledge and skills gained to manage diabetes (agree/strongly agree = 100%). The health literacy and disease knowledge education portion of the study intervention was particularly well-received, with 70–80% of the sample indicating that the nurse education and home visits were most helpful.

### Study outcome changes

Table 3 compares study outcomes at baseline, 12, and 24 weeks for the analytic cohort of 11 participants who completed the final assessments. At baseline, the mean HbA1c and fasting glucose were 9.3% (SD = 1.6) and 195.8 mg/dL (SD = 121.9), respectively. At 12 weeks, HbA1c and fasting glucose decreased with HbA1c decreasing by 0.4% (SD = 0.8) and fasting glucose decreasing by 22.9 mg/dL (SD = 126.4) corresponding to effect sizes of 0.27 and 0.19, respectively. Lipid and blood pressure outcomes also improved at 12 weeks with absolute effect sizes ranging from 0.14 to 0.33. These reductions from baseline did not continue at 24 weeks, however. The cohort had relatively high levels of diabetes literacy-reading (mean = 59.5, SD = 0.9) and low numeracy (mean = 1.7, SD = 1.2) at baseline. Diabetes reading scores decreased at 12 weeks but the trend reversed in the positive direction at 24 weeks. The mean improvements in numeracy were notable with relatively large effect sizes at both 12 and 24 weeks (0.46 and 0.54, respectively). For other psychosocial variables, the effect sizes ranged from 0.09 to 0.68 in absolute value. Of note, depression scores increased at 12 weeks but decreased from baseline value at 24 weeks with a small effect size of 0.10. Both self-efficacy and self-care increased from baseline to 12 weeks (effect sizes 0.57 and 0.29, respectively) but decreased slightly at 24 weeks. The participant reported social support and quality of life (VAS) increased over 24 weeks with effect sizes of 0.36 and 0.48, respectively.

**Table 3:** Outcome changes over 24 weeks (*N* = 11)

Variable	Mean (SD)			Mean change (SD) at 12 weeks^a^	Mean change (SD) at 24 weeks^b^	Effect size at 12 weeks^†^	Effect size at 24 weeks^††^
	Baseline	12 weeks	24 weeks				
Hemoglobin A1C, %	9.3 (1.6)	8.9 (1.8)	10.3 (3.2)	− 0.4 (0.8)	0.9 (3.6)	− 0.27	0.58
Fasting glucose, mg/dL	195.8 (121.9)	172.9 (52.9)	206.4 (109.0)	− 22.9 (126.4)	10.6 (124.1)	− 0.19	0.09
HDL cholesterol, mg/dL	46.5 (11.7)	44.7 (10.3)	44.3 (13.2)	− 1.7 (5.6)	− 2.2 (6.2)	− 0.15	− 0.19
LDL cholesterol, mg/dL	92.5 (25.2)	84.4 (19.4)	83.5 (23.0)	− 8.2 (13.1)	− 9 (23.7)	− 0.33	− 0.36
Triglycerides, mg/dL	129.1 (63.1)	113.4 (50.0)	141.1 (85.5)	− 15.7 (36.7)	12 (80.2)	− 0.25	0.19
Systolic BP, mmHg	130.0 (13.7)	127.5 (16.7)	130.5 (16.9)	− 2.4 (15.1)	0.6 (15.9)	− 0.14	0.04
Diastolic BP, mmHg	78.6 (7.1)	86.3 (34.2)	84.2 (8.3)	7.7 (33.2)	5.6 (7.8)	1.09	0.79
BMI	40.0 (10.4)	39.5 (9.8)	40.8 (9.7)	− 0.6 (1.9)	− 0.3 (3.1)	− 0.06	− 0.03
LAD	59.5 (0.9)	58.8 (2.0)	60 (0.0)	0.6 (2.0)	0.4 (0.8)	0.69	0.43
NVS	1.7 (1.2)	2.3 (1.4)	2.4 (1.5)	0.6 (1.4)	0.6 (1.1)	0.46	0.54
PHQ-9	6.0 (5.7)	7.0 (6.8)	6.1 (6.0)	0.6 (3.1)	− 0.6 (2.8)	0.09	− 0.10
DKT	6.0 (2.3)	7.2 (1.6)	7.5 (2.2)	1.2 (2.3)	1.6 (2.3)	0.52	0.68
DKT-insulin	1.0 (1.2)	1.4 (1.2)	1.2 (1.0)	0.4 (0.7)	0.2 (1.2)	0.31	0.15
SDSES	6.9 (1.9)	8.0 (1.5)	7.6 (1.8)	1.1 (2.0)	0.6 (2.0)	0.57	0.32
Self-care index	3.8 (1.4)	4.5 (1.4)	3.9 (0.8)	0.4 (1.6)	0.2 (1.1)	0.29	0.15
mMOS-SS	38.0 (12.5)	39.1 (9.3)	40.3 (8.7)	1.5 (15.2)	4.7 (16.1)	0.12	0.36
EQ-5	1.6 (0.4)	1.6 (0.4)	1.5 (0.4)	0.2 (1.6)	− 0.3 (1.3)	0.10	− 0.14
EQ-5-VAS	62.3 (25.6)	73.3 (23.8)	71.4 (22.0)	8.7 (14.5)	13.6 (25.7)	0.30	0.48

*BMI* body mass index, *HDL* high density lipoprotein, *LDL* low density lipoprotein, *DKT* Diabetes Knowledge Test, *LAD* Literacy Assessment in Diabetes, *REALM* Rapid Estimate of Adult Literacy, *NVS* Newest Vital Sign, *PHQ-9* Patient Health Questionnaire-9, *SDSES* Stanford Diabetes Self-Efficacy scale; *mMOS-SS* modified Medical Outcomes Study-Social Support Survey, *EQ-5* EuroQol-5, *EQ-5-VAS* EuroQol-5-visual analogue scale

^a^Mean change from baseline to 12 weeks

^b^Mean change from baseline to 24 weeks

^†^Mean change from baseline to 12 weeks divided by the standard deviation at baseline

^††^Mean change from baseline to 24 weeks divided by the standard deviation at baseline

## Discussion

To the best of our knowledge, PLAN 4 Success-Diabetes is the first intervention to integrate health literacy skills training with education to improve glycemic control among urban AA patients with diabetes. The theory-driven intervention program with a particular emphasis on health literacy training was well received by the pilot sample of inner-city AAs with uncontrolled diabetes. PLAN 4 Success-Diabetes also is the first to incorporate enhancing the home environment as an intervention component for safe physical activity planning in and around the home and dietary planning based on the food pantry and promoting home glucose monitoring. Of note, this method of doing a home visit in the community and individualized care is consistent with the American Diabetes Association’s Standards of Medical Care in Diabetes for optimal population health outcomes [30]. Home-based lifestyle intervention approaches have resulted in promoting healthy diet among adolescent moms [31], community-dwelling elders [32], and healthy diet and physical activity among older, obese/overweight cancer survivors [33, 34]. The involvement of community stakeholders in developing the intervention components helped to promote the credibility of PLAN 4 Success-DM as relevant to the target AA community.

Although this pilot study was not adequately powered to detect statistically significant outcome changes, the effect sizes estimated for the main study variables are encouraging. We achieved particularly large effect sizes in association with health literacy (both reading and numeracy), diabetes self-efficacy, and overall quality of life. HbA1c decreased among study participants at 12 weeks, although this change was not sustained at 24 weeks due, likely in part, to limited intervention delivery (average completed home visit = 1, average completed phone counseling = 3) because of limited resources to accommodate individual needs for scheduling home visits and counseling sessions outside of the schedules our interventionists could offer. Nevertheless, the results are promising and warrant further investigation to test the efficacy of the intervention with a sufficiently large sample size, especially given the high satisfaction of study participants with the study intervention.

We achieved a retention rate of 58% over 24 weeks in the pilot study sample of inner-city AAs. Other intervention studies involving mostly low-income patients with diabetes in a similar setting had retention rates ranging from 18% for a 12-month intervention involving 12 weekly education sessions followed by monthly follow-up sessions [35] to 100% for an 8-week group education intervention delivered weekly [36]. Another study including about two thirds of the study sample as AAs had a retention rate of 60% for a 20-week intervention with group education sessions delivered every 4 weeks [37]. Participant retention can be a recurring challenge in studies, particularly when the target sample is considered “hard to reach” based on social determinants of health. For example, according to the Transportation Research Board of the National Academies [38], 3.6 million Americans delay or miss medical care due to a transportation barrier each year. Based on our initial challenges with retention and relevant feedback from the community advisory committee, we shifted our transportation incentive from a $5 honorarium for each education session to direct transportation services. Six of the 11 participants in the final sample received Lyft services at some point during the study intervention. According to our brief assessment (data not shown), 100% of those who received this transportation service rated their experience as being highly satisfactory (mean = 10 on a 10-point scale). Half of them reported Lyft service affected their remaining in the study; three fourths said they would likely participate in a study that offers transportation. A recent systematic review [39] revealed that transportation interventions (e.g., offering bus passes, taxi/transport vouchers or reimbursement, arranging or connecting participants to transportation, and a free shuttle service) offered in combination with other tailored services improved patient outcomes such as cancer screening rates, chronic disease management, hospital utilization, and linkage and follow up to care. Future interventions targeting vulnerable groups of people such as inner-city low-income AAs should address transportation barriers—an important social determinant of health [40]—as part of the intervention to promote chronic disease management and patient outcomes.

The higher mean depressive symptom score observed in the participants who did not complete the study, compared with those who did is worth noting, though the difference was not statistically significant, due most likely to the small sample size. Depression has been recognized as an important factor associated with attrition from care. For example, a prospective cohort study [41] involving 610 HIV-infected African adults initiating antiretroviral therapy in Rwanda found that those with depression were two to four times more likely to experience attrition from care than those without depression. Similarly, in a RCT including non-Hispanic AA (*n* = 673) participants in the USA [42], early attrition among AA dropouts was associated with greater severity of clinician-rated depression. These findings suggest that identifying and effectively treating depression may help improve retention rates among AAs.

The major limitations of this pilot study were its small sample size and pre-post design without inclusion of a control group. In addition, the generalizability of this study is limited by inclusion of only AA participants from a low-income urban community, but we targeted this population because diabetes-related health disparities profoundly reported in AAs [1, 2]. Finally, one of the study instruments—diabetes self-care index—was created for the purpose of this study. Although our intent was to generate a brief yet comprehensive instrument to assess diabetes-related self-care activities, the use of a non-validated instrument such as ours may introduce challenges in that it does not allow cross-study comparisons.

## Conclusions

We achieved the attrition rate of 42% which was slightly higher than the 40% attrition rate identified a priori as the progression criterion. However, study participants found the PLAN 4 Success-Diabetes to be relevant and satisfactory, demonstrating the study intervention was highly acceptable. Our study findings provide important insights into promising intervention approaches in promoting glycemic control among inner-city AAs with diabetes. The positive effects of improved health literacy skills required for day-to-day management of diabetes may be more evident with a larger sample size than that of our pilot trial. Our findings also suggest some changes to optimize the protocols, before conducting a RCT. Specifically, future interventions should consider addressing social determinants of health such as transportation and depression as part of designing an intervention targeting low-income AAs with uncontrolled diabetes.

## Data Availability

The dataset used and analyzed during the current study are available from the corresponding author on reasonable request.

## Abbreviations

AAs: African Americans
PLAN 4 Success-Diabetes: Prevention through Lifestyle intervention And Numeracy-Diabetes
T2DM: type 2 diabetes mellitus
HbA1C: Hemoglobin A1C
LAD: Literacy Assessment in Diabetes
mMOS-SS: modified Medical Outcomes Study-Social Support Survey
PHQ-9: Patient Health Questionnaire-9
DSM-IV: Diagnostic and Statistical Manual of Mental Disorders, fourth edition
EQ: EuroQol
EQ-VAS: EuroQol visual analog scale
